# Choline kinetics in patients undergoing hypothermia treatment: a case observation in six cardiac arrest patients

**DOI:** 10.1186/cc9727

**Published:** 2011-03-11

**Authors:** T Schröder

**Affiliations:** 1 Charité-Universitätsmedizin Berlin, Germany

## Introduction

Lately it has been proven that mild therapeutic hypothermia (MTH) after cardiac arrest (CA) weakens the prognostic value of both neurological tests and serum markers, established before MTH was implemented [1-3]. Current prognostication and decision criteria have to be re-evaluated as well as new markers being necessary. Whole blood choline (WBCHO) and plasma choline (PLCHO) are promising new markers in cardiac arrest patients and they are under investigation as markers for global tissue ischemia [4-6]. It is unknown whether the recommended MTH treatment in patients after CA will influence choline levels. Therefore we analyzed choline kinetics in CA patients undergoing hypothermia treatment as a feasibility trial.

## Methods

All patients received MTH irrespective of the initial rhythm. Blood samples were taken on admission then again when reaching the therapeutic temperature of 33°C and after 12 hours of MTH at 33°C. All samples were stored at -80°C [4]. In order to determine the whole blood and plasma choline levels; high-pressure liquid chromatography combined with a mass spectrometer technique was used.

## Results

Six patients after cardiac arrest were analyzed in this feasibility trial. Four patients were male, two female. Median age was 66.5 years (interquartile range 57.5 to 82.25). Choline analyses revealed in five patients increased choline levels (> 10 μmol/l) on admission. Four patients showed a peak in both PLCHO and WBCHO when the 33°C target temperature during cooling was reached. Although MTH was maintained over 24 hours, in all cases the patients' choline levels decreased already after 12 hours of treatment to low or even subnormal concentrations.

## Conclusions

Both whole blood choline and plasma choline demon-strated a release pattern in patients after cardiac arrest undergoing hypothermia treatment. Larger studies have to evaluate the kinetics in detail and the potential prognostic implications of low or high choline levels in cardiac arrest patients.
